# STAT3 induces the expression of GLI1 in chronic lymphocytic leukemia cells

**DOI:** 10.18632/oncotarget.27884

**Published:** 2021-03-02

**Authors:** Uri Rozovski, David M. Harris, Ping Li, Zhiming Liu, Preetesh Jain, Taghi Manshouri, Ivo Veletic, Alessandra Ferrajoli, Prithviraj Bose, Phillip Thompson, Nitin Jain, Srdan Verstovsek, William Wierda, Michael J. Keating, Zeev Estrov

**Affiliations:** ^1^Department of Leukemia, The University of Texas MD Anderson Cancer Center, Houston, TX, USA; ^2^Division of Hematology, Davidoff Cancer Center, Rabin Medical Center, Petach Tiqva, and The Sackler Faculty of Medicine, Tel Aviv University, Tel Aviv, Israel

**Keywords:** CLL, STAT3, GLI1, apoptosis, transcription

## Abstract

The glioma associated oncogene-1 (GLI1), a downstream effector of the embryonic Hedgehog pathway, was detected in chronic lymphocytic leukemia (CLL), but not normal adult cells. GLI1 activating mutations were identified in 10% of patients with CLL. However, what induces GLI1 expression in GLI1-unmutated CLL cells is unknown. Because signal transducer and activator of transcription 3 (STAT3) is constitutively activated in CLL cells and sequence analysis detected putative STAT3-binding sites in the GLI1 gene promoter, we hypothesized that STAT3 induces the expression of GLI1. Western immunoblotting detected GLI1 in CLL cells from 7 of 7 patients, flow cytometry analysis confirmed that CD19+/CD5+ CLL cells co-express GLI1 and confocal microscopy showed co-localization of GLI1 and phosphorylated STAT3. Chromatin immunoprecipitation showed that STAT3 protein co-immunoprecipitated GLI1 as well as other STAT3-regulated genes. Transfection of CLL cells with STAT3-shRNA induced a mark decrease in GLI1 levels, suggesting that STAT3 binds to and induces the expression of GLI1 in CLL cells. An electromobility shift assay confirmed that STAT3 binds, and a luciferase assay showed that STAT3 activates the GLI1 gene. Transfection with GLI1-siRNA significantly increased the spontaneous apoptosis rate of CLL cells, suggesting that GLI1 inhibitors might provide therapeutic benefit to patients with CLL.

## INTRODUCTION

Chronic lymphocytic leukemia (CLL) is characterized by gradual accumulation of neoplastic B cells. Although CLL cells proliferate and die [1–3], constitutive activation of pro-survival pathways protects CLL cells from undergoing spontaneous apoptosis hence contributing to the steady buildup of the neoplastic clone [4–7].

The highly conserved Hedgehog signaling pathway, known to play a key role in embryonic development [8], was found to be active in Hedgehog-driven neoplasms such as neoplastic cells of the brain [9], lung [10] breast [11], and skin [12] and promote tumor cell survival. In CLL cells gene expression profiling detected high levels of the glioma associated oncogene-1 (GLI1) and GLI1 levels correlated with disease progression and unfavorable clinical outcome [13]. In addition, in approximately 10% of patients with CLL activating mutations of the GLI1 gene have been detected [14]. However, what induces the expression of GLI1 in CLL cells that do not carry GLI1 gene mutations is unknown.

In mammalian cells the signal transducer and activator of transcription 3 (STAT3) is ubiquitously expressed. Extracellular stimulation induces STAT3 phosphorylation, typically on tyrosine 705 residues. Phosphorylated STAT3 forms dimers, shuttles to the nucleus and functions as a transcription factor that triggers proliferation and activates anti-apoptotic programs [15]. In CLL cells STAT3 is constitutively phosphorylated on serine 727 residues [6, 16] and acetylated on lysine 685 residues [17]. Like phosphotyrosine STAT3, phosphoserine STAT3 binds and activates STAT3-regulated genes that provide CLL cells with a survival advantage [18–20]. Because sequence analysis detected putative STAT3 binding sites in the GLI1 gene promoter region, we wondered whether STAT3 induces the expression GLI1 in CLL cells.

## RESULTS

### CLL cells express high levels of GLI1 protein

**Figure 1:**
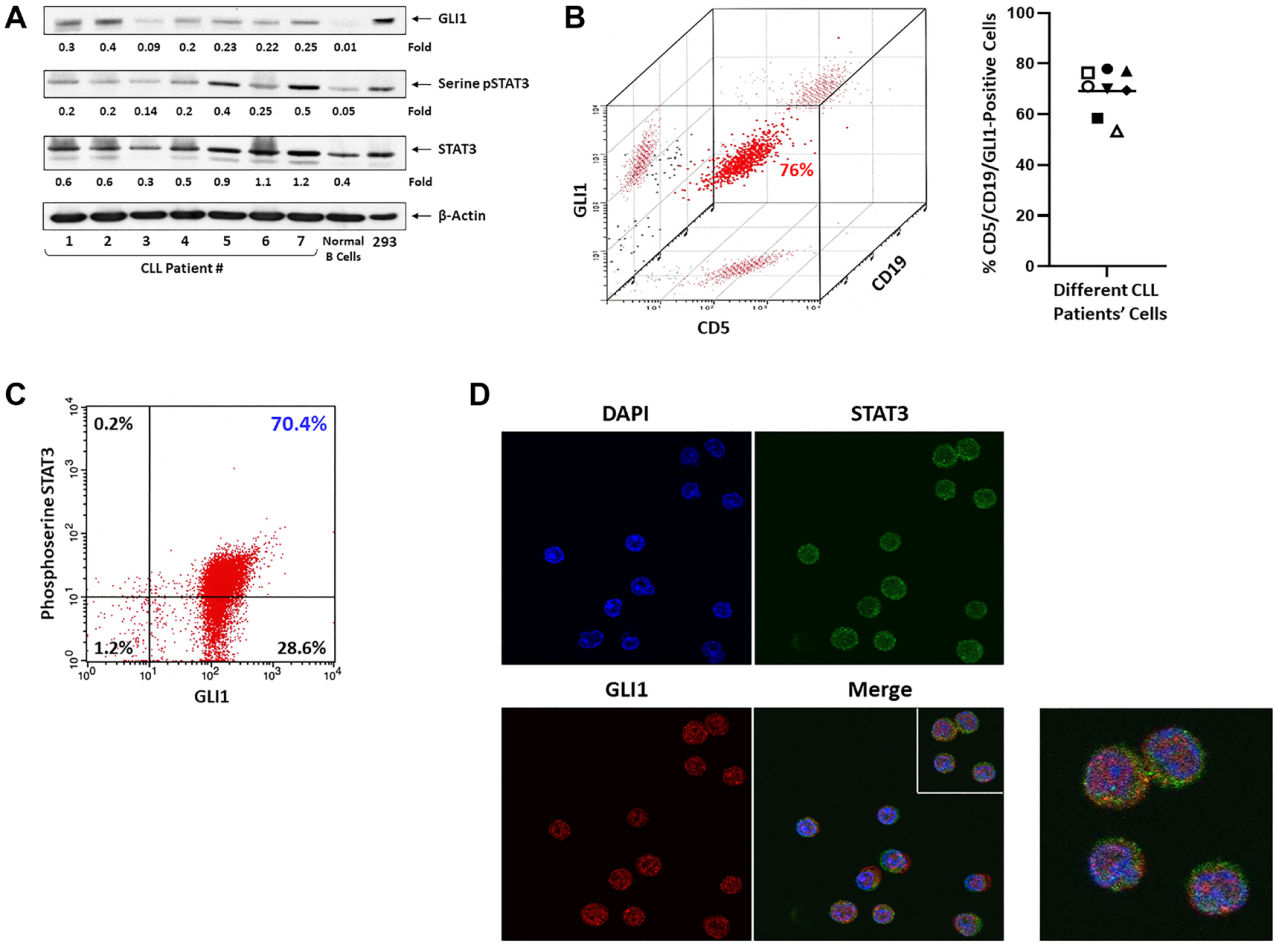
GLI1 protein detected in CLL cells but not in normal B cells. (**A**) Western blot analysis of CLL cells from 7 randomly selected patients and B cells from a healthy individual. As shown, GLI1, phosphoserine STAT3 and unphosphorylated STAT3 proteins were detected in CLL cell extracts of all patients. Conversely, GLI1 was undetected and the levels of phosphoserine STAT3 were very low in normal B cells. β-actin was used as loading control and densitometry units were normalized to actin levels in the corresponding lanes. HEK293 cell extract served as a positive control. (**B**) Flow cytometry analysis of CLL patient’s PB cells revealed that CLL cells co-express CD5 and CD19 and harbor intracellular Gli1. Left panel depicts representative data from one of 8 CLL patients. The right panel depicts the percent of cells co-expressing CD5/CD19/GLI1 in samples of 8 CLL patients. (**C**) Flow cytometry analysis of a different patient’s CLL cells showed that 70% of cells co-expressed intracellular phosphoserine STAT3 and GLI1. (**D**) Confocal microscopy images of fixed and permeablilized cells. As shown, intracellular STAT3 and GLI1 proteins are express in cytosol and the nucleus of the same CLL cells. DAPI (blue) was used to detect the cell nuclei. Original magnification ×100.

To assess the levels of GLI1 in CLL cells we performed western immunoblotting using CLL cell extracts from 7 randomly selected patients. Consistent with previously published data [6] GLI1 protein was detected in all CLL patients’ but not in normal B cell extracts and, as expected, total STAT3 and phosphoserine STAT3 were detected in CLL cells from all patients’ samples (Figure 1A). To further delineate these findings we performed flow cytometry analysis using peripheral blood (PB) low-density cells from 8 CLL patients. We found that 56% to 79% of cells that co-express CD5 and CD19, typically found in CLL cells, also express intracellular GLI1 (median co-expression: 69%) (Figure 1B). Then, to determine whether CLL cells co-express phosphoserine STAT3 and GLI1 we performed flow cytometry and confocal microscopy. Using flow cytometry we found that approximately 70% of CLL cells co-express intracellular GLI1 and phosphoserine STAT3 (Figure 1C), and using confocal microscopy we demonstrated the presence of both GLI1 and phosphoserine STAT3 in the cytoplasm and nucleus of CLL cells (Figure 1D).

### STAT3 co-immunoprecipitates GLI1 and STAT3-short hairpin (sh) RNA downregulates GLI1

We have previously demonstrated that STAT3 activates key components of pro-survival pathways in CLL cells [18, 19, 21, 22]. As shown above, CLL cells express high levels of the pro-survival GLI1 protein (Figure 1). Because STAT3 is constitutively activated in CLL cells and sequence analysis identified putative STAT3-binding sites in the GLI1 gene promoter, we wondered whether STAT3 activates GLI1 in CLL cells.

**Figure 2:**
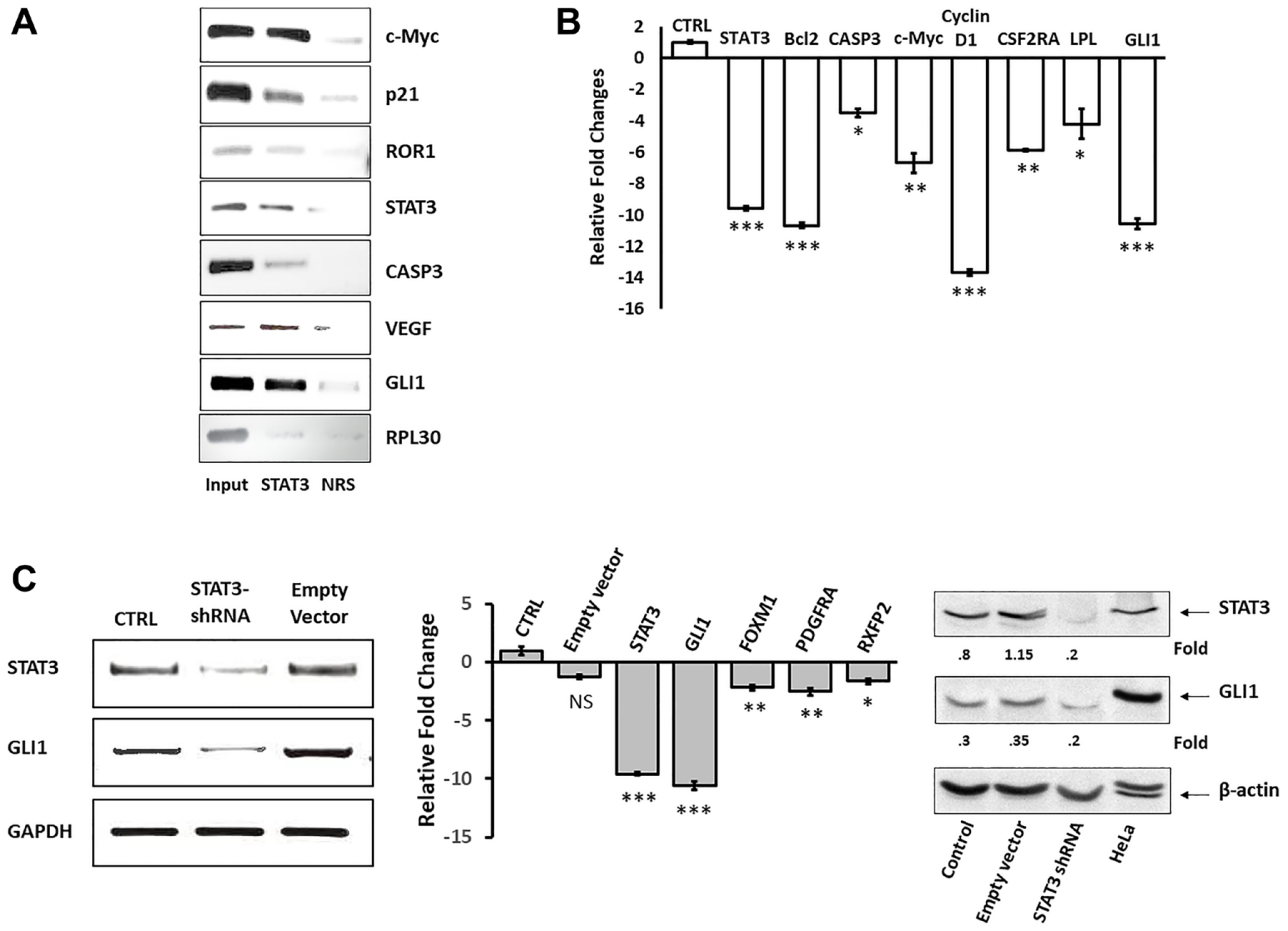
STAT3-shRNA downregulates GLI1 mRNA and protein levels in CLL cells. (**A**) To determine whether STAT3 binds GLI1 DNA in CLL cells we used ChIP. Protein extracts of CLL cells were incubated with or without anti-STAT3 antibodies and DNA was extracted from the precipitated chromatin fragments. Input denotes cross-linked and sonicated samples that were not immunoprecipitated and NRS (normal rabbit serum) denotes isotype control (negative control). As shown, anti-STAT3 antibodies co-immunoprecipitated DNA of the GLI1 and other STAT3-target genes. (**B**) The comparative C_T_ method was used to assess transcript levels in STAT3-shRNA infected cells at an infection efficiency of 45%. CTRL denotes infection with the empty lentiviral vector. Compared to control (CTRL), infection with STAT3-shRNA significantly downregulated transcript levels of GLI1 and other STAT3-regulated genes. The means ± standard deviations of 3 experiments are depicted. Transfected cells from 3 different patients yielded similar results. (**C**) CLL cells were transfected with STAT3-shRNA or with the empty lentiviral vector. As shown, mRNA levels of STAT3 and GLI1, assessed by conventional PCR (left panel) and qRT-PCR that also measured the levels of the GLI1-target genes FOXM1, PDGFRA and RFXP2 (middle panel), and STAT3 and GLI1 protein levels (right panel) were significantly downregulated in STAT3-shRNA transfected but not in empty vector-transfected CLL cells. HeLa cells served as control in the western blot analysis. ^***^ denotes *P* < 0.0001, ^**^ denotes *P* < 0.05, ^*^ denotes *P* < 0.07, and NS denotes statistically not significant compared to control. Expression levels were compared to the levels of STAT3 in CLL cells that were transfected with a lentiviral empty vector.

To answer this question we first used chromatin immunoprecipitation (ChIP) and found that STAT3 co-immunoprecipitated DNA of GLI1 as well as DNA of several STAT3-regulated genes including c-Myc, p21, ROR1, STAT3, caspase 3, and VEGF (Figure 2A). Then, to test whether STAT3 activates GLI1 in CLL cells, we transfected CLL cells with STAT3-shRNA. As shown in Figure 2B and 2C we found that STAT3-shRNA downregulated by 10-fold mRNA levels of GLI1 as well as mRNA levels of several STAT3-regulated genes including STAT3, Bcl2, caspase 3, c-Myc, cyclin D1, CSFRα, and LPL (Figure 2B). Furthermore, transfection of CLL cells with STAT3-shRNA, but not with the empty lentiviral vector, significantly downregulated the expression levels of STAT3, GLI1 and GLI1-target genes that are not STAT3-target genes, and of STAT3 and GLI1 protein levels (Figure 2C). Taken together, these data suggest that STAT3 binds to and activates the GLI1 gene in CLL cells.

### STAT3 binds and activates the GLI1 gene promoter

**Figure 3:**
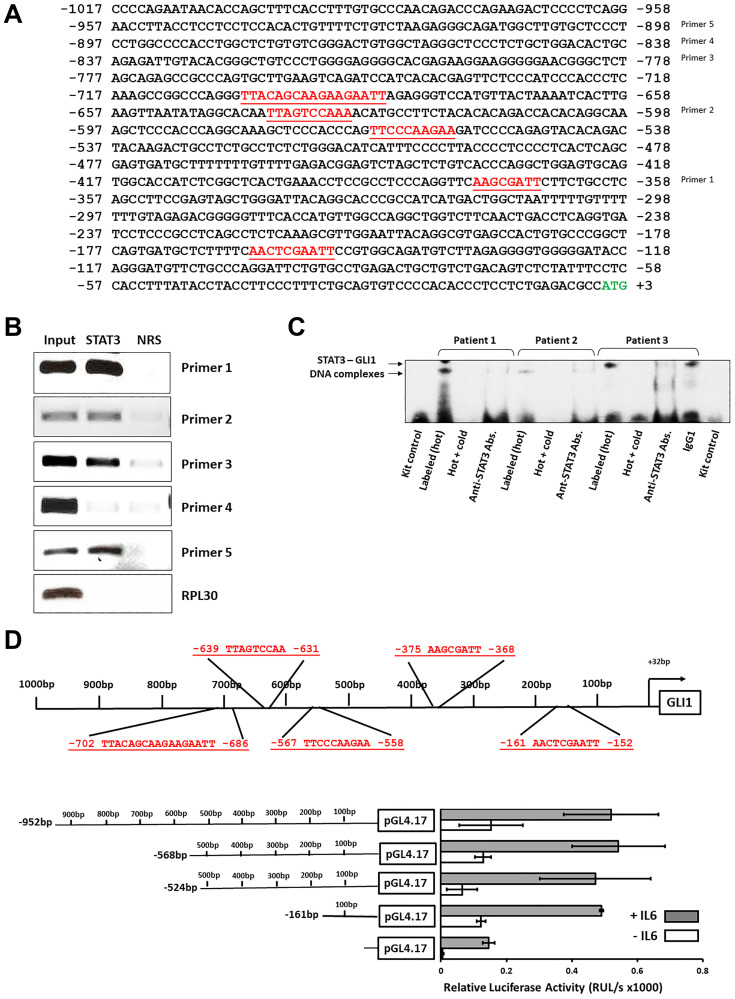
STAT3 binds and activates the GLI1 promoter in CLL cells. (**A**) Using the NCBI Web site https://www.ncbi.nlm.nih.gov/nuccore/NC_000012.12?report=genbank&from=57459785&to=57472451 we conducted sequence analysis and identified putative STAT-binding sites in the GLI1 gene promoter region. As shown, within 1000 bp upstream the *GLI1* transcription start codon (TSS) outlined in green, five interferon-γ activated sequences (GAS)-like elements (highlighted in red) constituting putative STAT3 binding sites. Right: Based on our sequence analysis we designed 5 probes, each designed to amplify a single putative STAT3 binding site. (**B**) Chromatin fragments of CLL cells were pulled down by anti-STAT3 antibodies and analyzed by PCR with primers directed at 5 putative STAT3 binding sites. As shown anti-STAT3 antibodies co-immunoprecipitated the DNA amplified by 4 different set of primers designated “1”, “2”, “3” and “5” but not by the set of primers designed to amplify putative STAT3 binding site located 631 to 639 bp upstream the *GLI1* TSS designated as “4”. Normal rabbit serum was used as isotype control and set of primers directed to amplify the *RPL30* promoter site were used as negative controls. Depicted is a representative figure from two different experiments with different patients’ cells. (**C**) Nuclear extract of CLL cells from 3 patients were incubated with biotinylated probe that included putative STAT3 binding site. EMSA showed that nuclear protein extracts from CLL cells of these patients bound the GLI1 DNA probe. The addition of excess unlabeled probe or binding to anti-STAT3 antibodies (but not IgG) attenuated the binding. (**D**) A luciferase assay confirmed that STAT3 activates *GLI1*. MM1 cells were transfected with truncated *GLI1* promoter region fragments harboring 0 (lower bar) to 5 (upper bar) harboring putative STAT3 binding sites (upper panel). MM1 cells were incubated with or without 20 ng/ml of IL-6, known to activate STAT3. As shown in the lower panel, in IL-6 stimulated cells luciferase activity significantly increased with each binding site added until it reached a plateau in a fragment that included 3 putative STAT3 binding sites. Data obtained from three different experiments are depicted.

Using the TFSEARCH database we identified five interferon-γ activated sequences (GAS)-like elements, considered putative STAT3 binding sites, within 1000 bp upstream the GLI1 gene start codon (Figure 3A). To determine which of those GAS-like elements are STAT3 binding sites we first used ChIP. Using ChIP we found that anti-STAT3 antibodies co-immunoprecipitated four different DNA fragments, each of which harbor GAS-like elements, suggesting that STAT3 bound 4 of the 5 putative STAT3-binding sits (Figure 3B). Then, to verify that STAT3 binds to the GLI1 gene promoter we used an electromobility shift assay (EMSA). Using a biotinylated DNA probe, corresponding to binding site –152 bp to –161 bp upstream of the GLI1 gene start codon, we found that the probe formed complexes with nuclear extracts of CLL cells obtained from 3 different patients. Excess unlabeled probe or anti-STAT3 antibodies attenuated the binding, thus confirming its specificity (Figure 3C). Together, the ChIP and EMSA findings implied that STAT3 binds the GLI1 promoter in CLL cells. To confirm that STAT3 activates the GLI1 gene promoter we used a luciferase assay. We transfected MM1 cells, in which interleukin (IL)-6 activates STAT3, with a luciferase reporter gene driven by fragments of the GLI1 promoter. We incubated the cells with or without IL-6 and assessed the transfected cells’ luciferase activity. We found that the degree of luciferase activity in IL-6-treated, but not IL-6-untreated, MM1 cells significantly increased with each added STAT3-binding site (Figure 3D). Taken together, these data suggesting that STAT3 binds to and activates the GLI1 gene.

### GLI1 protects CLL cells from apoptosis

**Figure 4:**
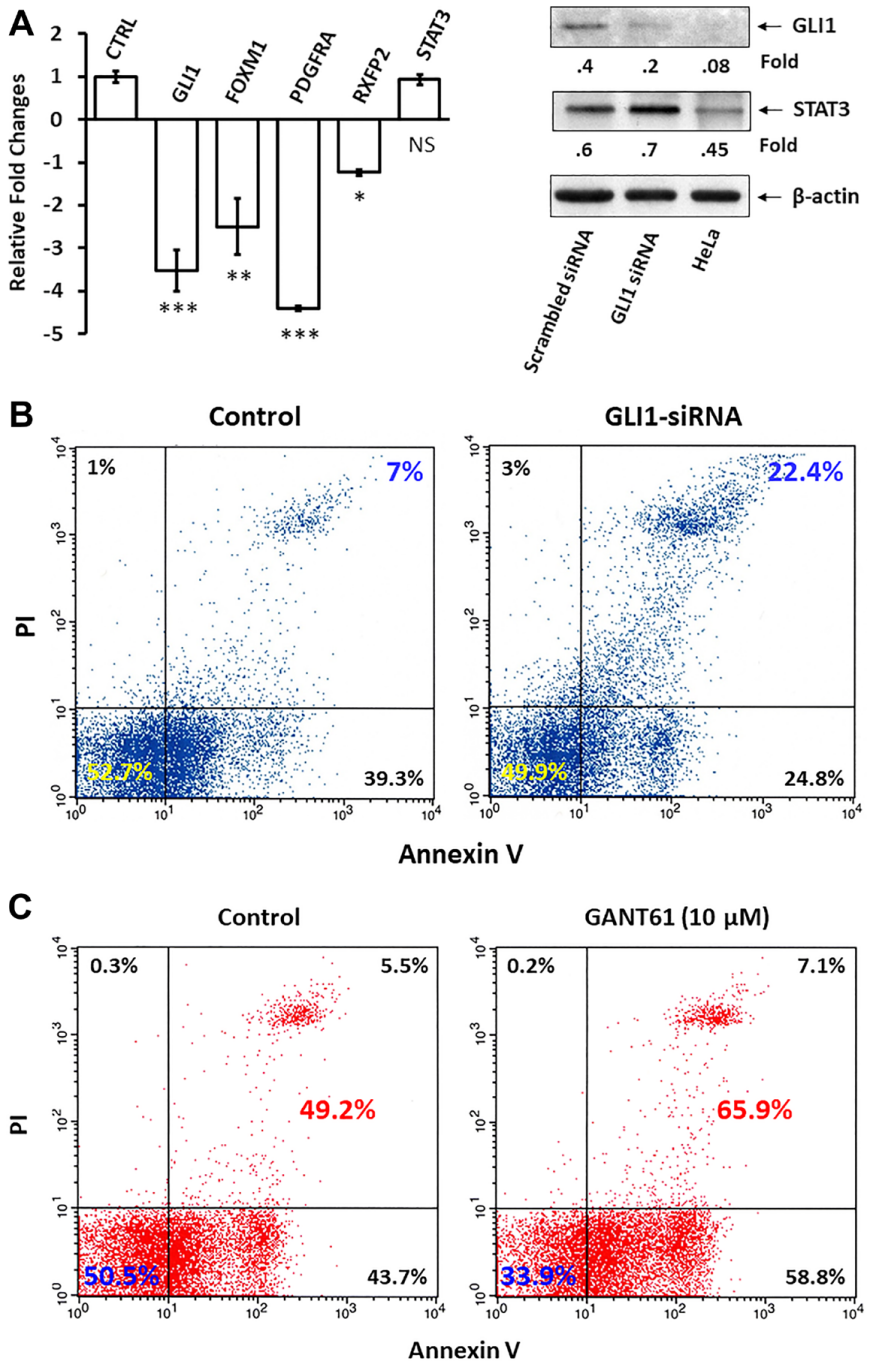
GLI1 protects CLL cells from apoptosis. (**A**) CLL cells were transfected with GLI1-siRNA or with an empty vector marked as CTRL (left bar). The comparative C_T_ method was used to compare the transcript levels of GLI1, STAT3 and GLI1-regulated genes Forkhead box M1 (FOXM1), Platelet-derived growth factor receptor-α (PDGRFA) and Relaxin family peptide receptor 2 (RFPR2). ^***^ denotes *P* < 0.0001, ^**^ denotes *P* < 0.001, ^*^ denotes *P* < 0.01, and NS denotes statistically not significant compared to control. (**B**) Flow cytometry analysis of CLL cells that were transfected with *GLI1*-siRNA or with GAPDH (transfection control). Apoptosis rate was assayed double staining with annexinV/PI. As shown the rates of apoptotic cells increased by approximately than 3-fold. (**C**) The apoptosis rates of CLL cells incubated overnight with or without 10 μM of the GLI1 inhibitor GANT61 are shown. Depicted are data from 3 different experiments.

Because STAT3 activates the GLI1 gene and GLI1 provides survival advantage to neoplastic cells, we sought to determine whether GLI1 contributes to the anti-apoptotic effect of STAT3 in CLL cells. To test this we transfected CLL cells with GLI1 small interfering (si) RNA. By using quantitative reverse transcription polymerase chain reaction (qRT-PCR) we found that transfection with GLI1-siRNA downregulated mRNA levels of GLI1 and the GLI-1-regulated genes Forkhead box M1 (FOXM1), Platelet-derived growth factor receptor-α (PDGRFA) and Relaxin family peptide receptor 2 (RFPR2) but not the levels of STAT3. Furthermore, GLI1-siRNA, but not scrambled siRNA, significantly downregulated GLI1 protein levels (Figure 4A). As expected, GLI1-siRNA significantly increased the spontaneous apoptosis rate of CLL cells (Figure 4B). Similarly, exposure of CLL cells to GANT61, an agent that selectively inhibits the transcription of GLI1, increased CLL cells’ apoptosis rate by 16.7% (Figure 4C), suggesting that GLI1 protects CLL cells from apoptosis.

## DISCUSSION

The Hedgehog pathway plays a critical role in embryonic life by enhancing the development of various organs including central nervous system pattering [23], tooth development [24] and limb formation [25]. Here we show that GLI1, a downstream effector of the Hedgehog pathway, is expressed in CLL cells, and that GLI1 expression is driven by STAT3.

The Hedgehog signaling pathway is not the only embryonic signaling pathway activated in CLL cells. We have recently reported that both Wnt5A, a downstream effector of the embryonic Wnt signaling pathway involved in cell fate, proliferation and migration, and its tyrosine-protein kinase transmembrane receptor ROR1 are commonly expressed in CLL cells. In addition, we found that like in GLI1, STAT3 induces the expression of Wnt5A and ROR1 [22, 26].

Reactivation of embryonic signaling pathways by either somatic mutations or epigenetic remodeling is a characteristic feature of cancer cells [27]. However, our data suggest that neither somatic mutations nor epigenetic remodeling is required for embryonic signaling pathway reactivation. STAT3, a master regulator of transcription, activates dormant embryonic pathways in the absence of activating mutations or epigenetic re-wiring.

Unlike the canonical ligand-induced phosphorylation of STAT3 operative in other cell types, in CLL cells STAT3 is constitutively phosphorylated on serine 727 residue [6, 16] by a unique cytosolic protein complex [28]. Like in CLL cells, in other cell types that express this isoform, cytosolic phosphoserine STAT3 translocates to the nucleus [29] and, similar to phosphotyrosine STAT3, binds GAS-like elements in the promoter of STAT3-regulated genes [19]. However, despite the similarities between phosphotyrosine and phosphoserine STAT3, different phosphorylation patterns may significantly alter the DNA binding affinity and activation dynamics leading to differential hierarchy of gene expression [30]. Our data suggest that the GLI1 gene, also activated by STAT3 in breast cancer cells [31], can be activated by either phosphoserine or phosphotyrosine STAT3. Although in CLL cells STAT3 is constitutively phosphorylated on serine residues, activation of the B cell receptor induces additional phosphorylation of STAT3 on tyrosine residues. Phosphorylation on two distinct sites increased the regulatory capacity of STAT3 and provided the cells with additional proliferation and survival advantage [32]. Hence, activation of GLI1 in B-cell proliferation centers might be more robust than in circulating CLL cells.

We found that similar to the levels of STAT3 and STAT3-regulated genes, the levels of GLI1 considerably varied between CLL cells of different patients. This patient-to-patient heterogeneity is likely the result of an alternative survival pathways that are activated during the time of clonal expansion.

Because GLI1 is overexpressed and provides CLL cells with survival advantage, GLI1 is a potential target for therapeutic intervention in CLL. Specific GLI1 inhibitors, such as GANT61, or commonly used agents with anti-GLI1 activity might increase the death rate of CLL cells. For example, arsenic trioxide, known for its activity in acute promyelocytic leukemia [33], was recently found to inhibit the transcription of GLI1 [34] and induce apoptosis of CLL cells [35]. Nevertheless, whether inhibition of the Hedgehog pathway by targeting GLI1 is an effective strategy to treat CLL, remain to be determined.

## MATERIALS AND METHODS

### Fractionation of CLL cells and normal B cells

We harvested CLL cells from the peripheral blood of treatment naïve patients who were treated at the University of Texas, MD Anderson Cancer Center. All study participants signed informed consent.

The patients’ clinical characteristics are depicted in Table 1. We used Ficoll-Hypaque 1077 (Sigma-Aldrich, St. Louis, MO, USA) to fractionate mononuclear cells and by flow we confirmed that at least 90% of the cells coexpressed CD19 and CD5, To isolate normal CD19^+^ B cells, normal donors’ PB low-density cells were fractionated using microimmunomagnetic beads and Miltenyi Columns (Miltenyi Biotec, San Diego, CA, USA) in accordance with the manufacturer’s instructions. More than 97% of the fractionated cells were CD19^+^ as assessed by flow cytometry.

**Table 1:** Baseline patient characteristics (*n* = 24)

Characteristic	Measure/Category	Overall
**Age, years**	Median (range)	60 (44–68)
**WBC** ×10^9^/L	Median (range)	27 (12–79)
**ALC** ×10^9^/L	Median (range)	23 (4–65)
**Rai stage**	(0, 1–2/3–4) (%)	(92/8)
**CD38**	≤ 30/> 30 (%)	79/21 (%)
**Zap-70**	Negative/Positive/unknown	46/21/33 (%)
**β2M (mg/L)**	(</≥ 4 mg/L)	96/4 (%)
**IGHV mutation**	(M/UM/unknown)	71/17/12 (%)
**FISH result, *n***	del17p/11q/T12/13q/Negative/unknown	1/1/2/9/3/8
**Karyotype, *n***	Diploid/Non-diploid/Complex/not done	14/6/0/4
**Survival status, (*n*)**	Alive/Dead	23/1

WBC, white blood cell count; ALC, absolute lymphocyte count; β2M, beta-2 microglobulin; M, mutated; UM, unmutated; FISH, fluorescence *in situ* hybridization.

### Western immunoblotting

Western blot analysis was performed as previously described [6] with the following antibodies: monoclonal anti-Gli1 (Novus Biologicals, Littleton, CO), monoclonal anti-total STAT3 (BD Biosciences Pharmingen, San Diego, CA), monoclonal anti-serine STAT3 (BD) and mouse anti-human β-actin (Sigma-Aldrich). Densitometry analysis was performed using an Epson Expression 1680 scanner (Epson America, Inc.). Densitometry values were normalized to β-actin.

### Flow cytometry

Cells were then stained with CD19 (BD Biosciences, San Jose CA), CD5 (BD Biosciences), GLI1 (Novus Biologicals), pSer-STAT3 (BD Biosciences) or with their corresponding isotypic control and analyzed on a FacsCaliber flow cytometer (BD Biosciences).

### Confocal microscopy

After fixing the cells with 2% paraformaldehyde we stained the cells with mouse anti-GLI1 and with mouse anti-STAT3 antibodies (BD Biosciences) for 1 hour, we incubated the cells with Alexa Fluor 488–labeled anti-rabbit and Alexa Fluor 647–labeled anti-mouse Abs for 30 minutes. Then, we viewed the cells with an Olympus FluoView 500 confocal laser-scanning microscope (Olympus America, Waltham, MA), and analyzed images by FluoView software (Olympus America).

### Chromatin immunoprecipitation (ChIP) assay

We performed chromatin immunoprecipitation (ChIP) assay by SimpleChIP Enzymatic Chromatin IP Kit (Cell Signaling Technology, Boston, MA) according to the manufacturer’s instructions using anti-STAT3 antibodies or rabbit serum (negative control). Antibody-bound protein-DNA complexes were eluted and subjected to polymerase chain reaction (PCR) analysis. The primers to amplify the human c-Myc promoter were F: 5-TGA GTA TAA AAG CCG GTT TTC-3 and R: 5-AGT AAT TCC AGC GAG AGG CAG-3, which generated a 63 bp product; to amplify the p21 gene promoter were F: 5-TTG TGC CAC TGC TGA CTT TGT C-3 and R: 5-CCT CAC ATC CTC CTT CTT CAG GCT-3 which generates a 303 bp product; to amplify the ROR1 promoter were F: 5-TTT GAG GAG TGT GGG GGA GGG-3 and R:5-GTT GAG AGG CTG CAG CAG AGG−3 which generated a 110 bp product; to amplify the STAT3 promoter were F: 5-CCG AAC GAG CTG GCC TTT CAT-3 and R: 5-GGA TTG GCT GAA GGG GCT GTA-3 which generated a 86 bp product; to amplify the CASP3 gene promoter were F: 5-TCC CAA CAG CCG GCT TAA-3 and R: 5-AAG AAG CCT GGT TTG GC-3 which generates a 65 bp product; to amplify the VEGF promoter were F: 5-CTT CTC CAG GCT CAC AGC TT-3 and R: 5- CCT GGA AAT AGC CAG GTC AG-3 which generated a 181 bp product. The primers to amplify fragments of the human GLI1 gene promoter regions encompassing γ-interferon activation sequence (GAS) binding sites were: F: -295(5′-TGT AGA GAC GGG GGT TTC AC-3′) and R: -96 (5′-CAG AAT CCT GGG CAG AAC TA-3’), which generates a 100 bp product that covers the -152 bp to –161 bp upstream of *GLI1* start codon; F: -526(5′-CTC TGC CTC TCT GGG ACA TC-3′) and R: -360 (5′-GGC AGA AGT ATC GCT TGA AC-3′), which generates a 166 bp product –375 bp to –368 bp upstream of *GLI1* start codon; F: -583 (5′-CAA AGC TCC CAC CCA GTT C-3′)and R: -479 (5′-CTG AGT GAG GGG AGG GGT-3′), which generates a 104 bp product –567 bp to –558 bp upstream of *GLI1* start codon; F: -744(5′-TCA CAC GAG TTC TCC CAT CC-3′) and R: -599(5′-TGC CTG TGT GGT CTG TGT GT-3′), which generates a 165 bp product –630 bp to –639 bp, –665 bp to –673 bp and –686 bp to –702 bp upstream of *GLI1* start codon; F: -1005(5′-ACC AGC TTT CAC CTT TGT GC-3′) and R: 816(5′-GGA CAG CCC GTG TAC AAT CT-3′), which generates a 189 bp –931 bp to –922 bp product upstream of *GLI1* start codon; and primers to amplify the human RPL30 gene were provided by Cell Signaling Technologies (Boston, MA, USA).

### Transfection of CLL cells with GFP-conjugated STAT3 short hairpin RNA (shRNA)

We transfected 293T cells with GFP-conjugated lentiviral STAT3 short hairpin RNA (STAT3 shRNA) or a GFP-conjugated empty lentiviral vector and with packaging vectors (pCMV delta R8.2 and pMDG generously provided by Dr. Giorgio Inghirami, (Department of Pathology, University of Torino, Italia) using the superfect transfection reagent (QIAGEN Inc.) as previously described [6].

### RNA purification and quantitative reverse transcription polymerase chain reaction (qRT-PCR)

To isolate RNA, we used RNeasy purification procedure (QIAGEN Inc., Valencia, CA, USA) and used NanoDrop spectrophotometer (ND-1000; NanoDrop Products, Wilmington, DE) to determine quality and concentration of the samples. Then, we Tperformed one-step qRT-PCR with the sequence detection system ABI Prism 7700 using TaqMan gene expression assays for *STAT3, BCL2, CASP3, c-Myc, Cyclin D1, CSF2RA, LPL, FOXM1, PDGFRA,* and *RXFP2* (all from Applied Biosystems) according to the manufacturer’s instructions.

Five hundred nanograms of total RNA was used in one- Relative quantification was performed using the comparative C_T_ method [36].

### Electrophoretic mobility shift assay (EMSA)

To perform EMSA we prepared nuclear extracts using NE-PER extraction kit (Thermo-Scientific Pierce; Rockford IL, USA). and incubated the extract with biotin-labeled DNA probes derived from the *GLI1* promoter sequence. Each probe designed to include one of the GAS-like elements in the *GLI1* promoter was synthesized by Sigma-Genosys (The Woodlands TX, USA). The probe (TTT TCA ACT CGA ATT CCG TG targets the GAS binding site –152 bp to –161 bp upstream of the *GLI1* start codon) was incubated for 30 minutes on ice. We separated each sample on a 5% polyacrylamide gel, transferred it, and fixed on the membrane via ultraviolet cross-linking. We used strepavidin-horseradish peroxidase (Gel-Shift Kit; Panomics Fremont CA, USA) to detect the probe and . a 7-fold excess of unlabeled cold probe As control. To confirm specificity of STAT3 binding we used anti-STAT3 antibodies (BD Biosciences) and mouse IgG1 (BD Biosciences).

### Transfection of MM-1 cells with GLI1 gene promoter fragments and luciferase assay

We transfected MM-1 cell by electroporation with 4 fragments of the *GLI1* promoter, each includes between 1 to 5 putative STAT3 binding sites. We selected MM-1 cells because in these cells extracellular signals such as interleukin (IL)-6 induce phosphorylation of STAT3. We assessed luciferase activity of unstimulated or IL-6 stimulated MM-1 cells after 24 h by a Dual-Luciferase Reporter Assay System (Promega) and a Sirius luminometer V3.1 (Berthold Detection Systems, Pforzheim, Germany).

### Transfection of CLL cell with GLI1 small interfering RNA (siRNA)

We mixed 10 ml of siPORT NeoFx transfection reagent diluted in 50 ml Opti-MEM I reduced serum medium with either GLI1 (siRNA), scrambled siRNA, FAM-labled human GAPDH or regaent control (Applied biosystems) at room temperature for 10 min. Then we added 1 × 10^7^ CLL cells suspended in 0.2 ml of Opti-MEM I and performed electroporation using the Gene Pulser Xcell Electroporation System (Bio-Rad Laboratories) and incubated the cells in RPMI 1640 (Thermo-Fisher Scientific) supplemented with 10% FBS for 24 h. To assess transfection efficiency, we used using a FACSCalibur flow cytometer (BD Biosciences).

Twenty micromolars human GLI1–small interfering RNA (siRNA) or scrambled siRNA, 20 mM FAM-labeled human GAPDH or reagent control (Applied Biosystems) were added to 10 ml siPORT NeoFX transfection reagent diluted in 50 ml Opti-MEM I reduced serum medium (Thermo-Fisher Scientific) and incubated at room temperature for 10 min. Next, the reagents were incubated at room temperature with 1 × 10^7^ CLL cells suspended in 0.2 ml of Opti-MEM I. After 1 h of incubation, electroporation was performed using the Gene Pulser Xcell Electroporation System (Bio-Rad Laboratories), and the cells were incubated in RPMI 1640 (Thermo-Fisher Scientific) supplemented with 10% FBS for 24 h. Transfection efficiency of the FAM-conjugated siRNA was assessed by flow cytometry using a FACSCalibur flow cytometer (BD Biosciences).

### Annexin/PI assay

The rates of cellular apoptosis of CLL cells transfected with GLI1-siRNA or incubated overnight with or without 10 μM of the GLI1 inhibitor GANT61 (Selleckchem, Houston, TX) were analyzed Annexin/PI assay(BD Biosciences) according to the manufacturer’s instructions.

## Abbreviations

CLL: Chronic lymphocytic leukemia
GLI1: Glioma associated oncogene-1
STAT3: Signal transducer and activator of transcription
(si) RNA: small interfering
(sh) RNA: short hairpin
ChIP: chromatin immunoprecipitation
EMSA: electromobility shift assay
IL: interleukin
FOXM1: forkhead box M1
PDGRFA: platelet-derived growth factor receptor-α
RFPR2: relaxin family peptide receptor 2
ROR: tyrosine-protein kinase transmembrane receptor
VEGEF: vascular endothelial growth factor
GAS: interferon-γ activated sequences
GFP: green fluorescent protein
BCL2: B-cell lymphoma 2
CSF2RA: colony stimulating factor 2 receptor subunit alpha
LPL: lipoprotein lipase
PI: propidium iodide
